# Simplified Chinese translation of 13 adult item banks from the Quality of Life in Neurological Disorders (Neuro-QoL)

**DOI:** 10.1186/s12913-018-3631-7

**Published:** 2018-10-30

**Authors:** Guanli Xie, Lidian Chen, Shanli Yang, Jing Tao, Chetwyn C. H. Chan, Allen W. Heinemann, David Cella, Jin-Shei Lai, Helena Correia, Alex W. K. Wong

**Affiliations:** 1 College of Rehabilitation Medicine, Fujian University of Traditional Chinese Medicine, 1 Huatuo Road, Minhou Shangjie, Fuzhou, 350122 Fujian China; 2 Traditional Chinese Medicine Rehabilitation Research Center of State Administration of Traditional Chinese Medicine of the P.R.C., Fuzhou, Fujian China; 3 Affiliated Rehabilitation Hospital of Fujian University of Traditional Chinese Medicine, Fuzhou, Fujian China; 4 Rehabilitation Medical Technology Joint National Local Engineering Research Center, Fuzhou, Fujian China; 5 Fujian Collaborative Innovation Center for Rehabilitation Technology, Fuzhou, Fujian China; 6 Department of Rehabilitation Sciences, The Hong Kong Polytechnic University, Hong Kong Special Administrative Region, China; 7 Department of Physical Medicine and Rehabilitation, Northwestern University Feinberg School of Medicine & Center for Rehabilitation Outcomes Research, Shirley Ryan Ability Lab (formerly Rehabilitation Institute of Chicago), Chicago, IL USA; 8 Department of Medical Social Science & Center for Patient-Centered Outcomes, Northwestern University Feinberg School of Medicine, Chicago, IL USA; 9 Departments of Medical Social Science & Pediatrics, Northwestern University Feinberg School of Medicine, Chicago, IL USA; 10 Department of Medical Social Science, Northwestern University Feinberg School of Medicine, Chicago, IL USA; 11 Program in Occupational Therapy and Department of Neurology, Washington University School of Medicine, 4444 Forest Park Ave, Campus Box 8505, St. Louis, MO 63108 USA

**Keywords:** Neuro-QoL, Translation, Item Bank, Cross-cultural validation

## Abstract

**Background:**

The Quality of Life in Neurological Disorders (Neuro-QoL) item banks evaluate and monitor the physical, mental, and social health of individuals with neurological conditions. Neuro-QoL items can be administered via short form or computerized adaptive testing. This paper describes the English-to-Simplified Chinese translation of 299 items from 13 adult item banks, which are publicly available.

**Methods:**

Items were translated according to the Functional Assessment of Chronic Illness Therapy (FACIT) method, including forward and backward translation, reconciliation, expert reviews, and cognitive debriefing with both general and clinical populations in China.

**Results:**

Most of the 299 Simplified Chinese items were well understood by the respondents. Revisions were made on a small number of items after cognitive debriefing. Although some difficulties were encountered in the translation process, all 13 item banks were linguistically validated with acceptable translations.

**Conclusion:**

All Chinese adult Neuro-QoL measures are linguistically equivalent to their English sources. Future work includes psychometric validation of these measures in order to create a final version of the item banks. The translation methodology used in this study can serve as a blueprint for researchers in other countries interested in translating the Neuro-QoL.

## Background

Neurological disorders can influence one’s health-related quality of life (HRQoL) in different domains. Precise measurement that evaluates different aspects of HRQoL provides a fuller understanding of the effects of disease or treatment on the patient [1, 2]. Nevertheless, many assessments have been developed to measure a single construct, which makes it difficult to obtain a comprehensive profile of individual patients [3–5]. Although some measures cover multiple domains, they are burdensome for patients because they are either too long or contain irrelevant questions [6]. The Quality of Life in Neurological Disorders (Neuro-QoL), a patient-reported outcome (PRO) measurement system covering multiple aspects of HRQoL of individuals with neurological disorders, was established to address these issues [7–9].

The Neuro-QoL project, sponsored by the United States (U.S.) National Institute of Neurological Disorders and Stroke (NINDS), aimed to develop a clinically relevant, bilingual (English & Spanish), and psychometrically robust PRO for adults and children with neurological disorders in the U.S. [7, 10]. Various domains of the Neuro-QoL were identified using extensive literature review, in-depth expert interviews, and focus groups. The final domains in the adult version were developed for five neurological conditions: stroke, multiple sclerosis, Parkinson’s disease, epilepsy, and amyotrophic lateral sclerosis. The psychometric properties of each domain were evaluated with item response theory (IRT) methods to enhance precision and efficiency [8, 11, 12]. The adult assessment is composed of 17 domains, 13 of which are tested and publicly available. IRT enables the creation of item banks (i.e., a collection of items measuring a single domain, such as depression). This approach allows for assessments to be administered in fixed-length short forms (SFs) or computerized adaptive tests (CATs) [11, 13–15]. The CAT approach allows for a tailored, computer-assisted assessment in which questions are determined by an individual’s response to previous questions. Thus, an individual’s domain level (the score on the instrument) is estimated based on the response given to each question. When the estimation reaches a pre-defined precision level, the computer stops asking questions and estimates the individual’s final domain level. Moreover, the Neuro-QoL uses standardized scores known as T-scores, which can be evaluated against a reference population. Using this metric, a score of 50 is the average of the reference population, with a standard deviation of 10. Thus, a score of 60 means that the individual is 1 standard deviation above the reference population. This common metric approach enables researchers to compare results of one individual to those of another. Additional details and updates including definitions, translation, administration, and score interpretations are available at https://www.assessmentcenter.net/.

In addition to the original English version, 12 adult and 11 child item banks were translated into Spanish for use in the U.S. and in Spanish-speaking countries or regions (e.g., Puerto Rico, Mexico, Argentina, Colombia, Spain). In 2015, the Simplified Chinese Neuro-QoL working group adopted and implemented the Neuro-QoL in China. The working group involved a group of outcome scientists, neurorehabilitation and physical medicine professionals, and language translation coordinators. This paper describes the linguistic translation and cultural adaptation of 13 Neuro-QoL item banks for use by adults in China. These item banks were chosen because they were publicly available, and translation authorization was granted by the Neuro-QoL project PI and co-author (D.C.) for this validation process.

## Methods

The 13 item banks have a total of 299 items (Table 1). They were translated according to the Functional Assessment of Chronic Illness in Therapy (FACIT) translation methodology, which employs a universal approach to translation and cross-cultural validation [16, 17]. It consists of forward and back translations, multiple reviews, and pilot testing with cognitive debriefing. These methods ensure semantic, conceptual, and cultural equivalence between the original English and the Simplified Chinese versions. The present study protocol was approved by the Ethics Committee of The Affiliated Rehabilitation Hospital of Fujian University of Traditional Chinese Medicine (2016KY-023-01). Informed consent was obtained from all participants included in the study. Below we describe the seven steps we took to translate the Neuro-QoL.

- Step 1: Forward Translation

**Table 1:** Thirteen translated Neuro-QoL adult item banks

English	Simplified Chinese	Number of Items in Each Bank
Upper Extremity Function (fine motor, ADL)	上肢功能(精细活动、日常生活活动能力)	20
Lower Extremity Function (mobility)	下肢功能(移动能力)	19
Fatigue	疲劳	19
Sleep Disturbance	睡眠紊乱	8
Depression	抑郁	24
Anxiety	焦虑	21
Stigma	歧视	24
Positive Affect and Well-being	积极情感和幸福感	23
Emotional and Behavioral Dyscontrol	情绪和行为失控	18
Cognitive Function	认知功能	28
Communication	交流	5
Ability to Participate in Social Roles and Activities	参与社会角色和活动的能力	45
Satisfaction with Social Roles and Activities	社会角色和社会活动的满意度	45

The original source (i.e., English-language Neuro-QoL item banks) was translated independently by two bilingual translators (native Mandarin speakers) with experience in PRO research. They were instructed to give a response to each item and use simple language to capture the meaning of the original item rather than a literal, word-by-word translation. Item definitions were provided to clarify the intended meanings of the concepts.

- Step 2: Reconciliation

A third Mandarin-speaking professional translator reconciled the two forward translations. He was instructed to avoid slang terms and region-specific expressions. He selected the best translation or gave an alternate version, if necessary, to convey the clearest meaning of the items.

- Step 3: Back Translation

A fourth bilingual translator who was blind to the original English version back-translated the reconciled version into English.

- Step 4: Compare Back Translation to Source

The working group consisting of one physician and three occupational and physical therapists working in neurorehabilitation units compared the back translation with the original English source. The back translation should reflect the same meaning as the original source. Coherence and influence of the back translation were reviewed. Discrepancies were identified for discussion during the expert review.

- Step 5: Expert Review

Seven bilingual experts in neurorehabilitation and PRO research examined all previous steps and evaluated the acceptability of the translated items. They were asked to evaluate the equivalence, relevance, and representativeness of each item independently and provide alternative translations. For each of the discrepancy items identified in Step 4, they were asked to give a proper translation and provide the justification. Afterward, all experts discussed unacceptable items to reach a consensus.

- Step 6: Harmonization, Quality Control, and Proofreading

The working group evaluated experts’ recommendations to make the pre-final translation. They assessed the equivalence and consistency across items and verified that documentation of the decision-making process was complete. Two proofreaders independently examined items for any remaining linguistic issues. The working group refined and documented these items accordingly.

- Step7: Cognitive Interviews

Cognitive interviews were completed with 20 Mandarin-speaking individuals to determine whether the respondents accurately understood the intended meaning of each item. The average age of respondents was 58 years old (SD = 13.8, range: 21–78). Ten respondents were from the general population living near the University, and 10 were inpatients of The Affiliated Rehabilitation Hospital of Fujian University of Traditional Chinese Medicine (3 diagnosed with cerebral infarction, 3 diagnosed with cerebral hemorrhage, 2 diagnosed with traumatic brain injury, and 2 diagnosed with spinal cord injury). Men comprised 60% of the general and clinical samples. The majority of participants in the sample were married (general = 6, clinical = 7) and completed primary education or had no formal education (general = 7, clinical = 7). The majority of the clinical sample (60%) was unemployed or retired, whereas the majority of the general sample (60%) was employed full-time. We did not test the readability of the translated items. Nevertheless, we assume that all items could be comprehended by those with low literacy because the majority of the sample (70%) only completed primary education or had no formal education.

The goal of cognitive interviews was to examine how the participant interpreted each item or responded in terms of comprehension of what the item was asking. To achieve this goal, each respondent completed all Neuro-QoL items independently. Then, the interviewer reviewed each item stem and item response with the respondent and began the interview using a debriefing script. Probes were used to elicit feedback about the item phrasing, response category, instruction, and recall period. In some instances, respondents were asked why they selected a specific response and were invited to offer alternative item wordings. After each interview, the interviewer completed a summary statement with all comments for each item. The translation team reviewed all comments to determine whether any revisions should be made.

## Results

### Analysis and finalization

Respondents’ comments in the cognitive interviews were analyzed. Translations of the items were revised when the item difficulty or respondents’ comments revealed a potential misunderstanding of the intended meaning. Overall, most items obtained an acceptable Simplified Chinese translation, and no item bank stood out as being more problematic than another. An example of the entire translation process for one item is provided in Table 2.

**Table 2:** Example of the translation process of one item from the Ability to Participate in Social Roles and Activities item bank

NQPRF1	Item Wording
Original English	I have to limit my hobbies or leisure activities
Forward 1	我不得不限制自己的爱好和休闲活动
Forward 2	我不得不限制我的爱好和休闲活动
Reconciliation	我不得不限制我的业余爱好和休闲活动
Back Translation	I have to limit my hobbies and recreation activities
FACIT	1. Do you agree with the words chosen by the Reconciliation (REC) translation? 2. Also, we should confirm that the current translation is acceptable.
Reviewer 1 (Chinese)	REC is OK
Reviewer 2 (Chinese)	Suggestion:我必须限制我的业余爱好和休闲活动. “不得不” indicates that you must do something for compelling reason. This is a written language rather than a usual expression. I am afraid that it is hard to understand for any person with low education.
Reviewer 3 (English)	Suggestion: 我要限制我的业余爱好和休闲活动 “不得不” emphasizes that I cannot do that because of limitations of my body function. Instead, the word “要” indicates that I cannot paticipate in any activities, either because of my limitation or because I don’t want to.
Reviewer 4 (Chinese)	Suggestion:我必须/要限制我的业余爱好和休闲活动. “不得不” is a double-negative word. I think that most Chinese people do not understand. Conversely, I suggest “必须” and “要,” which are more acceptable and understandable by most people.
Reviewer 5 (Chinese)	The translation is OK. However, “不得不” is not often used in the Chinese context.
Reviewer 6 (English)	REC looks good for me.
Reviewer 7 (English)	I suggested that we should get some feedback from our patients to decide if either one of these three words: “不得不,” “必须,” or “要” would work better.
Translation working team comments	Reviewers agreed that REC is the final translation; however, they recommended keeping these terms and asked participants during cognitive interviews.
Proofreading	我**不得不**限制我的业余爱好和休闲活动 我**必须**限制我的业余爱好和休闲活动 我**要**限制我的业余爱好和休闲活动
Post-test final	After cognitive review, the final translation was confirmed: 我必须限制我的业余爱好和休闲活动. Most participants in the cognitive interview suggested that “必须” is idiomatic Chinese. Four participants reported that “不得不” is not often used in China, and they have difficulty understanding it. Three participants considered “不得不” difficult to understand for persons with low education. Three participants suggested that “要” is unsuitable because it does not describe the limitation the person faces to participate in social activities.

We also identified numerous challenges in the translation process, including:

1. *Past tense representation*: the original English version asks about respondents’ past behaviors by use of the past tense. Simplified Chinese items did not distinguish between present and past tense. Specific timing words were used to reflect past tense, such as“了.”
2. *Influence of idiomatic Chinese*: this was evident in the understanding of the term “I felt.” Mandarin speakers could interpret the term as “我觉得”or “我感到.” Although the two phrases do not have a significant conceptual difference, the latter was chosen because it is used more often in everyday communication.
3. *Subtle semantic difference of possible translations*: in Simplified Chinese, no substantial distinction is made between “nervous” and “tense,” whereas both words are used in the original English Anxiety item bank to indicate different nuances in the experience of anxiety. The translation of these words in Simplified Chinese literally means “紧张.” To clarify the subtle difference in expression, “nervous” was translated into “精神紧张,” and “tense” was translated into “身体紧绷,” given that the word “nervous” describes a mental representation of the anxious experience, whereas “tense” describes a somatic representation of that experience.
4. *Ambiguity of translated items*: for example, an item in the original English Fatigue item bank, “I need to sleep during the day,” was not understood by the Mandarin-speaking Chinese respondents because time to rest after lunch is a constitutional right, and daytime napping is not uncommon; thus, “I need to sleep during the day,” does not necessarily indicate fatigue. Instead, an elaborated phrase, “I am so tired,” was added to clarify the intended meaning: “我累得需要在白天睡觉” (“I am so tired that I need to sleep during the day”).
5. *Morphology of language:* the order of words was considered inappropriate if the sentence was directly translated verbatim from the original source. For example, the original English item, “In most ways my life is close to my ideal,” was translated as “我的人生在大多数情况下接近我的理想” (“My life is close to my ideal in most ways”). Articles (i.e., “a,” “an,” and “the”) were not translated.

## Discussion

This study completed the first translation of 13 adult Neuro-QoL item banks from English to Simplified Chinese. It also presented the first completed large-scale Neuro-QoL translation performed outside of the U.S. We followed a rigorous, multi-step translation methodology that follows international guidelines for the linguistic validation of PROs for non-English-speaking populations [18]. This methodology incorporates input from bilingual translators and includes pre-testing with cognitive debriefing to ensure that items are conceptually equivalent to the English source and are culturally appropriate to a Mandarin-speaking Chinese population. Although we encountered some difficulties throughout this process, we ultimately achieved cultural equivalence for these items.

While present findings support Chinese measures that are linguistically equivalent to the original English versions, the extent to which they are psychometrically comparable remains to be determined. The Simplified Chinese items have been administered to a calibration sample of over 1000 Mandarin-speaking adults in China. Calibration of the Simplified Chinese items by following standardized psychometric validation methods with the original English version [8], such as confirmatory factor analysis (CFA), IRT-based item calibration, and differential item functioning (DIF), is underway. After the calibration is considered final, all Simplified Chinese fixed-length short forms will be available for use and download via Health Measures, the official website for the Neuro-QoL and other measurement systems (https://www.assessmentcenter.net/).

We recommend the use of Neuro-QoL item banks in future Chinese studies after we confirm that the psychometric properties of the Simplified Chinese item banks are comparable to those of the original versions. Future research on these validation procedures will increase confidence in their use.

Use of the Neuro-QoL has clear benefits over many traditional questionnaires developed by the classical test theory approach. First, the Neuro-QoL item banks were psychometrically tested using modern statistical (IRT) methods. This approach enables assessment with smaller measurement error (better precision) and can reduce sample size requirements in studies. Neuro-QoL measures are also responsive to change, making them suitable for use in routine clinical practice and for benchmarking [11, 12, 19]. Furthermore, the development of the Neuro-QoL included extensive participant and expert input, increasing the acceptability of clinical utilization [8, 20, 21]. The scoring of the Neuro-QoL item banks is expressed on a common metric (mean T-score of 50 and standard deviation of 10), which facilitates comparisons of findings across patients and between studies [20, 22]. To facilitate the use of Neuro-QoL item banks in China, we recommend the use of the CAT platform. Increasing technological access and high-speed internet will enhance the feasibility of using CAT in outcome assessments for both clinical research and practice. However, the best way to implement CAT administration of the Neuro-QoL in various clinical populations and settings deserves further investigation. The present study is our initial step to ensure the conceptual and semantic equivalence between Simplified Chinese and English measures. Validation studies of these translated measures are ongoing. After the psychometric properties of translated measures are tested, cross-cultural validation of the Chinese- and English-language measures will continue for international comparisons of HRQoL studies.

## Conclusion

Neuro-QoL items have been linguistically validated with acceptable translations to Simplified Chinese. The intended meanings and concepts of these translated instruments are the same when compared to the original English versions. After we confirm the psychometric properties of these translated measures in future studies, it is expected that the Neuro-QoL may be used worldwide, which will facilitate international comparison research in areas of neurology and rehabilitation. Second, the translation methodology described in this paper will provide a template for researchers in other countries interested in translating the Neuro-QoL and other outcome measures.

## Abbreviations

CAT: Computerized adaptive test
CFA: Confirmatory factor analysis
DIF: Differential item functioning
FACIT: The Functional Assessment of Chronic Illness in Therapy
HRQoL: Health-related quality of life
IRT: Item response theory
Neuro-QoL: The Quality of Life in Neurological Disorders
P.R.C.: The People’s Republic of China
PRO: Patient-reported outcome

## References

[1] Webster K, Cella D, Yost K (2003). The Functional Assessment of Chronic Illness Therapy (FACIT) measurement system: properties, applications, and interpretation. Health Qual Life Outcomes.

[2] Cella D, Nowinski CJ (2002). Measuring quality of life in chronic illness: the functional assessment of chronic illness therapy measurement system. Arch Phys Med Rehabil.

[3] Polinder S, Haagsma JA, Belt E, Lyons RA, Erasmus V, Lund J, Beeck EFV (2010). A systematic review of studies measuring health-related quality of life of general injury populations. BMC Public Health.

[4] Polinder S, Haagsma JA, Klaveren D, Steyerberg EW, EFV B (2015). Health-related quality of life after TBI: a systematic review of study design, instruments, measurement properties, and outcome. Popul Health Metrics.

[5] Nichol AD, Higgins AM, Gabbe BJ, Murray LJ, Cooper DJ, Cameron PA (2011). Measuring functional and quality of life outcomes following major head injury: common scales and checklists. Injury.

[6] Tulsky DS, Kisala PA, Victorson D, Tate D, Heinemann AW, Amtmann D, Cella D (2011). Developing a contemporary patient-reported outcomes measure for spinal cord injury. Arch Phys Med Rehabil.

[7] Cella D, Nowinski CJ, Peterman A, Victorson D, Miller D, Lai JS, Moy C (2011). The neurology quality-of-life measurement initiative. Arch Phys Med Rehabil.

[8] Gershon RC, Lai JS, Bode R, Choi S, Moy C, Bleck T, Miller D, Peterman A, Cella D (2012). Neuro-QOL: quality of life item banks for adults with neurological disorders: item development and calibrations based upon clinical and general population testing. Qual Life Res.

[9] Perez L, Huang J, Jansky L, Nowinski CJ, Victorson D, Peterman A, Cella D (2007). Using focus groups to inform the neuro-QOL measurement tool: exploring patient-centered, health-related quality of life concepts across neurological conditions. J Neurosci Nurs.

[10] Correia H, Pérez B, Arnold B, Wong AW, Lai JS, Kallen M, Cella D (2015). Spanish translation and linguistic validation of the quality of life in neurological disorders (Neuro-QoL) measurement system. Qual Life Res.

[11] Quatrano LA, Cruz TH (2011). Future of outcomes measurement: impact on research in medical rehabilitation and neurologic populations. Arch Phys Med Rehabil.

[12] Tulsky DS, Carlozzi N, Cella D (2011). Advances in outcomes measurement in rehabilitation medicine: current initiatives from the National Institutes of Health and the National Institute on Disability and Rehabilitation Research. Arch Phys Med Rehabil.

[13] Cella D, Lai JS, Nowinski C, Victorson D, Peterman A, Miller D, Bethoux F, Heinemann AW, Rubin S, Cavazos JE (2012). Neuro-QOL brief measures of health-related quality of life for clinical research in neurology. Neurology.

[14] Kozlowski AJ, Cella D, Nitsch KP, Heinemann AW (2015). Evaluating individual change with the Quality of Life in Neurological Disorders (Neuro-QoL) short forms. Arch Phys Med Rehabil.

[15] Victorson D, Peterman A, Bode R, Buono S, Mueller A, Moy C, Cella D (2015). Development and clinical validation of a new item Bank and short form of emotional and behavioral Dyscontrol for major neurological disorders: results from the neuro-QOL study. J Neurol Disord Stroke.

[16] Eremenco S, Cella D, Arnold BJ (2005). A comprehensive method for the translation and cross-cultural validation of health status questionnaires. Eval Health Prof.

[17] Wild D, Eremenco S, Mear I, Martin M, Gawlicki M, Hareendran A, Wiklund I, Chong LY, Houchin C, Rv M (2009). Multinational trials-recommendations on the translations required, approaches to using the same language in different countries, and the approaches to support pooling the data: the ISPOR patient-reported outcomes translation and linguistic validation good. Value Health.

[18] Beaton DE, Bombardier C, Guillemin F, Ferraz MB (2000). Guidelines for the process of cross-cultural adaptation of self-report measures. Spine.

[19] Fayers PM (2007). Applying item response theory and computer adaptive testing: the challenges for health outcomes assessment. Qual Life Res.

[20] User Manual for the Quality of Life in Neurological Disorders (Neuro-QoL) Measures, Version 2.0 [http://www.healthmeasures.net/images/neuro_qol/Neuro-QOL_User_Manual_v2_24Mar2015.pdf]. Accessed 4 Feb 2017.

[21] Final Report of the Neuro-QoL Study [http://www.healthmeasures.net/images/neuro_qol/NeuroQOL-Final_report-2013.pdf]. Accessed 4 Feb 2017.

[22] Neuro-QoL Technical Report [http://www.healthmeasures.net/images/neuro_qol/Neuro-QoL_Manual_Technical_Report_v2_24Mar2015.pdf]. Accessed 4 Feb 2017.

